# Chemical Composition and Biological Activities of *Artemisia pedemontana* subsp. *assoana* Essential Oils and Hydrolate

**DOI:** 10.3390/biom9100558

**Published:** 2019-10-02

**Authors:** Paula Sainz, María Fe Andrés, Rafael A. Martínez-Díaz, María Bailén, Juliana Navarro-Rocha, Carmen E. Díaz, Azucena González-Coloma

**Affiliations:** 1 Instituto de Ciencias Agrarias, Consejo Superior de Investigaciones Científicas, 28006 Madrid, Spain; paula.sainz.sotomayor@gmail.com (P.S.); mafay@ica.csic.es (M.F.A.); 2 Departamento de Medicina Preventiva, Salud Pública y Microbiología, Facultad de Medicina, Universidad Autónoma de Madrid, 28029 Madrid, Spain; rafael.martinez@uam.es; 3 Departamento de Farmacia y Biotecnología, Facultad de Ciencias, Universidad Europea de Madrid, 28670 Villaviciosa de Odón, Madrid, Spain; maria.bailen@universidadeuropea.es; 4 Centro de Investigación y Tecnología Agroalimentaria de Aragón, Unidad de Recursos Forestales, 50059 Zaragoza, Spain; jnavarroro@aragon.es; 5 Instituto de Productos Naturales y Agrobiología, Consejo Superior de Investigaciones Científicas, 38206 La Laguna, Tenerife, Spain; celisa@ipna.csic.es

**Keywords:** *Artemisia pedemontana* subspecies *assoana*, experimental cultivation, essential oil, hydrolate, antitrypanosomal, phytomonacidal, antiplasmodial, insect antifeedant, phytotoxic, antifungal, nematicidal

## Abstract

Given the importance of the genus *Artemisia* as a source of valuable natural products, the rare plant *Artemisia pedemontana* subspecies *assoana,* endemic to the Iberian Peninsula, has been experimentally cultivated in the greenhouse and aeroponically, to produce biomass for essential oil (EO) extraction. The chemical composition of the EOs was analyzed, and their plant protection (insects: *Spodoptera littoralis*, *Rhopalosiphum padi*, and *Myzus persicae*; plants: *Lactuca sativa* and *Lolium perenne*; fungi: *Aspergillus niger*; and nematode: *Meloidogyne javanica*) and antiparasitic (*Trypanosoma cruzi*, *Phytomonas davidi*, and antiplasmodial by the ferriprotoporphyrin biocrystallization inhibition test) properties were studied, in addition to the hydrolate by-product. The EOs showed a 1,8-cineole and camphor profile, with quantitative and qualitative chemical differences between the cultivation methods. These oils had moderate insect antifeedant, antifungal, and phytotoxic effects; were trypanocidel; and exhibited moderate phytomonacidal effects, while the hydrolate showed a strong nematicidal activity. Both EOs were similarly antifeedant; the EO from the greenhouse plants (flowering stage) was more biocidal (antifungal, nematicidal, and phytotoxic) than the EO from the aeroponic plants (growing stage), which was more antiparasitic. The major components of the oils (1,8-cineole and camphor), or their 1:1 combination, did not explain any of these effects. We can conclude that these EOs have potential applications as insect antifeedants, and as antifungal or antiparasitic agents, depending on the cultivation method, and that the hydrolate byproduct is a potent nematicidal.

## 1. Introduction

The genus *Artemisia* (Asteraceae family) has been widely studied from a phytochemical point of view. *Artemisia* species produce essential oils [1] and have a broad spectrum of bioactivity, including antibacterial [2,3], antiviral [4], antiparasitic [5,6,7,8,9], antifungal [10], nematicidal [11], and insecticidal [12,13], and overall, contribute a great deal to the plant defensive properties. However, some species have been poorly studied, mostly because of their lack of abundance or distribution [14]. One of such species is *Artemisia pedemontana* (Bent), a whitish-lanate perennial aromatic shrub with a circummediterranean disjunct distribution [15]. In the Iberian Peninsula, the endemic subspecies *A. pedemontana* subsp. *assoana* (Willk.) Rivas Mart. grows in disturbed grazed land in a continental climate at 900–2000 m [16].

This plant subspecies has been subjected to a few phytochemical studies. There is one report on the essential oil composition of wild *A. pedemontana* subsp. *assoana* from two populations [17]. Additionally, two sesquiterpene lactones have been identified from organic extracts of its aerial parts [18], while the roots yielded flavones, coumarins, diacetylenic spiroketal enolethers, and n-akyl *p*-coumarates [19]. There are no additional studies on the bioactivity (pharmacological or other) of *A. pedemontana* subsp. *assoana* extracts or components.

As part of an ongoing valorization of rare species from the genus *Artemisia* from the Iberian Peninsula, this work studied the essential oil composition of experimentally cultivated *A. pedemontana* subsp. *assoana* (in a greenhouse without environmental control, and in an aeroponic system with a controlled environment).

Given the importance of *Artemisia* species as a source of antiparasitic and biocidal agents [14], the antiparasitic effects of these essential oils were tested against epimastigotes of *Trypanosoma cruzi*, the etiologic agent of the Chagas disease, and promastigotes of *Phytomonas davidi*, a plant trypanosomatid that causes several diseases in crops of great importance [20]. The antimalarial potential of these oils was tested in vitro using the ferriprotoporphyrin (FP) IX biocrystallization inhibition [21,22]. The plant protection effects included insect antifeedant (*Spodoptera littoralis*, *Myzus persicae* and *Rhopalosiphum padi*), antifungal (*Aspergillus niger*), phytotoxic (*Lactuca sativa* and *Lolium perenne*), and nematicidal (*Meloidogyne javanica*) activities.

## 2. Materials and Methods 

### 2.1. Plant Material and Cultivation

*A. pedemontana* subsp. *assoana* seeds were collected from a wild population (Campillo de Aragón, Spain, GPS coordinates 41°06′31.7″N 1°50′39.7″W, 1050 m of altitude) and germinated in a growth chamber (25 °C, 70% RH with a photoperiod of 16:8 h (light (L):dark(D)).

The seedlings were grown in a substrate composed of three parts of sand and one of peat, and were cultivated in a greenhouse without environmental control. A selected plant was used for the vegetative reproduction, and these plants were used in the aeroponic and greenhouse experimental cultivation. Plants of 10–15 cm height, kept in a growth chamber at the environmental conditions described above, were transferred to an aeroponic chamber (Apollo 3 system: 33 plants, 240 L, 1750 mm × 1350 mm × 750 mm) located in a growth chamber (25 °C, 70% RH with a photoperiod of 16:8 h (L:D). The plants were pulverized for 15 min every 2 h with water supplemented with 0.2 g/L Nutrichem (20:20:20 of N:P:K—Miller Chemical and Fertilizer Crop, Hanover, PA, USA) and 0.03 % H_2_O_2_ (33% *w/v* Panreac Química SLU, Castellar del Vallès, Barcelona, Spain).

The plant aerial parts were collected at the flowering stage from the greenhouse, and periodically, 20–30 cm plants were selected from the aeroponic chamber at growing stage, dried (26 °C for five days in an air-forced ventilated stove), and grounded prior to extraction.

### 2.2. Essential Oil Extraction

Hydrodistillation was performed using a Clevenger-type apparatus with an extraction chamber, separated according to the method recommended by the European Pharmacopoeia (http://www.edqm.eu/en/Homepage-628.html). Each extraction was carried out in triplicate with 100 g of dried aerial plant parts for 2 h. The hydrolate (aqueous phase) was decanted from the essential oil collected in the condensation section of the Clevenger, and was filtered. The total volumes obtained were 2.1 and 1.2 mL of greenhouse and aeroponic EO (0.7% and 0.4% yield, respectively) and 20 mL of hydrolate. The EOs were dried over anhydrous sodium sulfate and stored at 4–6 °C until the chemical analysis.

### 2.3. Gas Chromatography–Mass Spectrometry Analysis

The essential oils were analyzed by gas chromatography-mass spectrometry (GC-MS) using a Shimadzu GC-2010 gas chromatograph coupled to a Shimadzu GCMS-QP2010 Ultra mass detector (electron ionization, 70 eV, Duisburg, Germany) and equipped with a 30 m × 0.25 mm i.d. capillary column (0.25 µm film thickness) Teknokroma TRB-5 (95%) dimethyl-(5%) diphenylpolisiloxane. The working conditions were as follows: split ratio (20:1), injector temperature 300 °C, temperature of the transfer line connecter to the mass spectrometer 250 °C, initial column temperature 70 °C, then heated to 290 °C at 6 °C/min. Electron ionization mass spectra and retention data were used to assess the identity of the compounds by comparing them with those of the standards or those found in the Wiley 229 Mass Spectral Database. The relative amounts of individual components were calculated based on the GC peak area (flame ionization Detector, FID, response), without using a correction factor. The relative standard deviation (calculated as SD/average × 100) for the % peak area (three injections/sample) was ≤1%. Electron ionization mass spectra and retention data were used to assess the identity of the compounds by comparing them with those found in the Wiley 229 Mass Spectral Database.

### 2.4. Antiparasite Bioassays

The human parasite *Trypanosoma cruzi* Y-strain, and the plant parasite *Phytomonas davidi* ATCC^®^ 30287TM strain, were used to test antiparasitic effects on the epimastigote and promastigote forms, respectively. *T. cruzi* epimastigotes and *P. davidi* promastigotes were grown axenically at 28 °C in liver infusion tryptose (LIT) supplemented with 10% heat-inactivated fetal calf serum (FCS; Gibco, Dublin, Ireland).

#### 2.4.1. *Trypanosoma cruzi* Susceptibility Assay

The activity on the epimastigote forms of *T. cruzi* was evaluated on cultures in a LIT medium supplemented with 10% heat-inactivated (FCS). The parasites in the logarithmic growth phase (8–10 × 10^6^ epimastigote/mL) were distributed in 96-well flat-bottom plates (90 µL of culture/well). Essential oils and compounds (1,8-cineole and camphor from Sigma-Aldrich Quimica SL, Madrid, Spain) were tested in triplicate at several concentrations (EOs at 800, 400, 200, and 100 µg/mL; compounds at 100, 10, and 1 µg/mL) for 72 h. Nifurtimox (Bayer AG, Monheim am Rhein, Germany) was used as the positive control, and the parasite viability was analyzed by a modified 3-(4,5-dimethylthiazol-2-yl)-2,5-diphenyltetrazolium bromide (MTT) colorimetric assay method [7]. Data were analyzed with Statgraphics statistical analysis software (Centurion XVIII, Statgraphics Technologies, Inc., The Plains, VA, USA) using one-way analysis of variance (ANOVA) and least significant difference (LSD) test (*p* < 0.05) analyses.

#### 2.4.2. *Phytomonas davidi* Susceptibility Assay

A new bioassay has been developed to evaluate the activity of the EOs against promastigote forms of *P. davidi,* cultured in a LIT medium supplemented with 10% heat-inactivated (FCS). Parasites in the logarithmic growth phase (5 × 10^4^ promastigotes/mL) were distributed in 96-well flat-bottom plates (90 µL of culture/well). The essential oils and compounds were tested in triplicate at several concentrations (EOs at 800, 400, 200 and 100 µg/mL; compounds at 100, 10 and 1 µg/mL) for 24 h. The parasite viability was analyzed by a modified MTT colorimetric assay method using Menadione instead of phenazine methosulfate (PMS) as the electron-coupling agent. Briefly, after 24 h, 10 µL of the solution of 15 mg of MTT, and 0.5 mg Menadione in 3 mL PBS were added to each well. This was incubated for 75 min for the reduction of MTT to occur, and 100 µL sodium dodecyl sulphate was added to dissolve the formazan crystals obtained as a result of the reduction of MTT. The absorbance was read on a spectrophotometer at 603 nm. The activity was calculated as in the epimastigote assay, and submitted to the same statistical analysis described before.

#### 2.4.3. Ferriprotoporphyrin (FP) IX Biocrystallization Inhibition Test (FBIT)

This bioassay was performed to evaluate the inhibition of FP biocrystallization in presence of the essential oils and the pure compounds [22]. The bioassay was carried out in a non-sterile 96-well plate flat-bottom at 37 °C for 18–24 h. The EOs and pure compounds were tested at several concentrations (EOs at 10, 5, and 2.5 mg/mL; and compounds at 100, 50, and 25 µg/mL). Chloroquine biphosphate (Sigma-Aldrich) was used as the reference drug. A series of solutions were added to the plate, namely: 0.5 mg/mL of hemin chloride (Sigma-Aldrich) freshly dissolved in dimethylsulphoxide (DMSO) (50 µL), 100 µL of 0.5 M sodium acetate buffer (pH 4.4), and 50 µL of EOs or compounds dissolved in DMSO. After 18–24 h, the plates were centrifuged at 3000 rpm for 5 min, and the supernatant was discarded. The remaining pellet was resuspended in 200 µL of DMSO so as to remove the unreacted FP. The plate was centrifuged again and the supernatant was discarded. The pellet was dissolved in 150 µL of 0.1 M NaOH, and the absorbance measured at 405 nm. The percentage of inhibition of the FP biocrystallization was calculated as follows:

Inhibition (%) = 100 × [(OD control − OD treatment)/OD control](1)

### 2.5. Insect Bioassays

*Spodoptera littoralis*, *Myzus persicae,* and *Rhopalosiphum padi* colonies were reared on an artificial diet [23], bell pepper (*Capsicum annuum*), and barley (*Hordeum vulgare*) plants, respectively, and maintained at 22 ± 1 °C, >70% relative humidity with a photoperiod of 16:8 h (L:D) in a growth chamber.

The bioassays were conducted with newly emerged *S. littoralis* L6 larvae (2/plate, 10 plates) or *M. persicae/R. padi* adults (10/box, 20 boxes). The feeding or settling inhibition (%FI or %SI) were calculated as %FI = [1-(T/C)] x 100, where T and C are the consumption of the treated and control leaf disks, respectively, or as %SI = [1-(%T/%C)] × 100, where %C and %T are the percentage of aphids settled on the control and treated leaf disks, respectively [24]. The antifeedant effects (%FI/%SI) were analyzed for significance by the non-parametric Wilcoxon signed rank test. The EC_50_ values (effective dose to obtain 50% feeding inhibition) were determined for the EOs and pure compounds with %FI/%SI values >70% from a linear regression analysis (Statgraphics statistical analysis software).

### 2.6. Phytotoxic Activity

The experiments were conducted with *Lactuca sativa* cv. Teresa (Fitó, Selva de Mar, Barcelona, Spain), and *Lolium perenne* (Batlle, Molins de Rei, Barcelona, Spain) seeds, as described [25]. Briefly, 2.5 cm diameter filter paper with 20 µL of the test solution (10 µg/µL for EOs, 5 µg/µL for pure compounds) were placed on 12-well plates (Falcon, Corning, NY, USA) and then 500 µL H_2_O/well and 10/5 seeds (*L. sativa*/*L. perenne* pre-soaked in distilled water for 12 h) were added. The covered plates were placed in a plant growth chamber (25 °C, 70% RH, 16:8 L:D), and the germination was monitored for six days. At the end of the experiment, the root/leaf length was measured (25 plantlets randomly selected for each experiment and digitalized with the application ImageJ 1.43 (http://imagej.nih.gov/ij/)).

### 2.7. Antifungal Bioassays

The essential oils and the major compounds were tested against *Aspergillus niger* (donated by Dr. K. Leiss, Wageningen University). The antifungal activity was measured by an MTT colorimetric spore germination inhibition test [26]. Afterwards, the subculture from the potato dextrose agar (PDA) plates’ conidial suspensions were prepared. For susceptibility testing, 1.5 × 10^4^ conidia per well (counted in a Neubauer chamber) were seeded into flat-bottom 96-well plates in 200 µL of morpholine-propanesulfonic acid (MOPS)-buffered RPMI medium (Sigma Aldrich). The plates were incubated for 24 h at 30 °C. Amphotericin B (5 µg/mL) was used as a positive control and DMSO (1%) as a negative control (solvent and drug free control). After incubation, the spore germination was measured by a colorimetric method using the dye MTT. The medium (RPMI, 25 µL) with 5 mg/mL of MTT and menadione 1 mM was added to each well and incubated at 37 °C for 3 h. The content of each well was removed, and 200 µL of isopropanol with 5% of HCl (1 M) were added to each well to extract the dye. After 30 min of incubation at room temperature and gentle agitation, the optical density (OD) was measured at 490 nm. Data were analyzed with Statgraphics statistical analysis software (Centurion XVIII) using ANOVA and least significant difference (LSD) test (*p* < 0.05) analysis.

### 2.8. Nematicidal Bioassays

A field-selected *Meloidogyne javanica* population from Barcelona, Spain, was maintained on tomato plants (*Solanum lycopersicum* L. var. *Marmande*) in pot cultures at 25 ± 1 °C, >70% relative humidity. The egg masses of *M. javanica* were handpicked from infected tomato roots two months after the inoculation of the seedlings. Second-stage juveniles (J2) were obtained by incubating the egg masses in a water suspension at 25 °C for 24 h.

#### 2.8.1. In Vitro Effect on Juveniles

The experiments were carried out in 96-well microplates, as described previously [11]. The EOs were tested at an initial concentration of 1 mg/mL in DMSO-Tween (0.5% Tween20 in DMSO), and the hydrolate was tested without dilution (100%). Serial dilutions (1:2) were carried out when needed. All of the treatments were replicated four times, and the number of dead juveniles was recorded after 72 h. The data were determined as percentage of mortality, corrected according to Scheider–Orelli’s formula. The data were analyzed using ANOVA and least significant difference (LSD) test (*p* < 0.05) analysis. Effective lethal doses (LC_50_) were calculated by Probit analysis (Statgraphics statistical analysis software, Centurion XVIII).

#### 2.8.2. In Vitro Effect on Egg Hatching

Three egg masses of uniform size were washed with sterilized distilled water and transferred to a 24-well plate containing 400 µL of the hydrolate (HAasA). The egg masses in the sterilized distilled water were used as the controls. Each experiment was replicated four times. The plates were covered to prevent evaporation, and were incubated in the darkness at 25 °C. After five days, the hatched J2s were counted and the test solutions were replaced with sterilized distilled water. The egg masses were monitored for four weeks at weekly intervals, until hatching was complete in the control [11]. The relative hatch suppression rate (compared with the controls) was calculated.

#### 2.8.3. Effect on Juvenile Infection Capacity

Three-week-old tomato seedlings (susceptible variety, Marmande, France) were transplanted into 5-cm-diameter clay pots filled with 10 mL of quartz sand. The seedlings were individually inoculated with 180–200 J2 untreated (control), or treated with hydrolate (HAasA) at a sublethal dose (LC_50_), and were incubated for one week in a growth chamber (25 ± 2 °C, 60% RH, and 16:8 L:D). The seedlings were then removed and the roots were stained with acid fuchsine [11]. Juveniles within the roots of each individual seedling were counted by examining the entire root system under a stereomicroscope. The experiment consisted of six replicas, and was repeated two times. The relative percentages of the J2 penetration (treated vs. untreated) were calculated in order to obtain inhibition rates of J2 infectivity [11]. The Chi-square test was used to calculate the statistically significant difference between the proportions of the control juveniles (untreated) and treated juveniles that penetrated the tomato roots (Statgraphics statistical analysis software, Centurion XVIII).

## 3. Results and Discussion

### 3.1. Biomass Production and Essential Oil (EO) Composition

**Figure 1:**
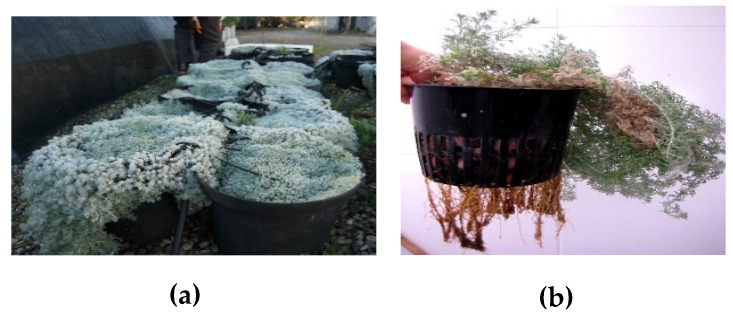
*Artemisia pedemontana* subsp. *assoana* pictures from both cultivation methods, namely: (**a**) plants growing in a greenhouse (AasG); (**b**) plant growing in an aeroponic system (AsA).

Two cultivation methods were used for the biomass production of *A. pedemontana* subsp. *Assoana*, namely: greenhouse and aeroponic systems. The greenhouse plants (AasG) grown without environmental control were morphologically similar to plants from wild populations (shorter and thicker), and flowered after the winter vegetative stage, while the aeroponic plants (AasA), grown with environmental control, remained in the growing stage, did not flower, and grew faster (Figure 1). 

The essential oils (EOs) from the greenhouse and aeroponically cultivated *A. pedemontana* subsp. *assoana* yielded 0.7% and 0.4% (plant dry weight), respectively. The phenology of the samples (greenhouse in flowering stage and aeroponic in growing stage) could explain the difference in essential oil yield.

Table 1 shows the chemical composition of the essential oils. The major components were 1,8-cineole (22.8–25.8%) and camphor (32.4–44.0%). Quantitative differences were observed between the essential oils from the greenhouse and aeroponic plants. The monoterpene camphor was more abundant in the greenhouse population (AasG), while 1,8-cineole was more abundant in the oil from the aeroponic plants (AasA). Terpinen-4-ol (8.8%) was the third major component of the AasG EO, followed by borneol (4.8%), 1-α-terpineol (3.6%), and camphene (1.8%), while the AasA EO had *p*-cymene (7.4%), terpinen-4-ol (5.8%), camphene (5.3%), and borneol (2.8%) as the main components.

There is only one report describing the chemical composition of the essential oils of two populations of *A. pedemonana* subsp. *assoana*. The authors suggested two different chemotypes, camphor and 1,8-cineole, and davanone [17]. Our results agree with the first chemotype (camphor and 1,8-cineole). Both monoterpenes are very common in the chemical composition of *Artemisia* sp. essential oils. Species like *A. annua, A. fragans, A. longifolia*, and *A. ludoviciana* are also rich in these two monoterpenes [1].

The qualitative differences between these oils included α-terpinene (0.9%), γ-terpiene (1.7%), sabinene (2.5%), and sabinene isomer (1.5%), only present in AasA; and linalool (1.6%), bornyl acetate (0.9%), and nerolidol (0.8%), only found in the AasG EO.

### 3.2. Antiparasitic Effects

The antiparasitic activity of the essential oils, their major compounds (camphor and 1,8-cineole), and their 1:1 combination are shown in Table 2. Among the essential oils, the one from the aeroponic population (AasA) was more active than the oil from the greenhouse plants against both parasites. The *T. cruzi* epimastigotes were more sensible to the effect of these EOs than the *P. davidi* promastigotes. The fact that *P. davidi* is a plant parasite could explain this selective effect. On the other hand, none of the major compounds (1,8-cineole and camphor) in their 1:1 mixture showed an antiparasitic activity. Similarly, none of the essential oils or the pure compounds tested showed an inhibition of the FP biocrystallization in the FBIT test (Table S1 in Supplementary Material). 

*Artemisia* sp. essential oils have been reported as being trypanocidal [14], specifically, essential oil from cultivated *A. absinthium* [5,7]. However, this is the first report on the trypanocidal activity of *A. pedemontana* subsp. *assoana* essential oil. A recent study reported 1,8-cineole as being inactive against *T. cruzi*, while compounds such as *p*-cymene, linalool, β-pinene, and γ-terpinene had strong effects [6]. Moreover, binary combinations of these compounds showed synergistic effects [6]. These compounds are also present in *A. pedemontana* subsp. *assoana* essential oils in different concentrations, and could explain the antiparasitic effects described here. 

Several species of *Phytomonas* have been reported as being responsible of plant diseases in crops of economic importance, such as coffee and palm trees [20], with the removal of the infected plants being the only control available. Synthetic compounds such as triazolo-pyrimidine complexes have been reported as being active against *Phytomonas* sp. [27,28,29], and the natural alkaloids tomatine and tomatidine against *P. serpens* [30]. However, this is the first report of the activity of essential oils against promastigotes of *Phytomonas* sp.

### 3.3. Insect Antifeedant and Biocidal Effects

We tested the effects of the essential oils from *A. pedemontana* subsp. *assoana* against herbivorous insects, plants, nematodes, and fungi, to have an overall idea of the plant defensive properties allocated to its EO, in addition to identifying the effects of their potential use in plant protection.

Table 3 shows the insect antifeedant effects of *A. pedemontana* subsp. *assoana* EOs, their major components 1,8-cineole and camphor, and a mixture (1:1) of both. The EO from the greenhouse population (AasG) was antifeedant to *S. littoralis* at the highest concentration tested, and showed moderate–low activity against the aphid *R. padi*. The aeroponic plant (AasA) EO was a moderate antifeedant to *M. persicae* and *R. padi*. The different antifeedant effects observed for *A. pedemontana* subsp. *assoana* EOs could be related to the quantitative and qualitative differences found in their chemical composition. Furthermore, the major EO components tested (1,8-cineole and camphor) did not show significant antifeedant effects alone or in a 1:1 mixture. These results indicate that the insect antifeedant activity could be modulated by the minor compounds of the EO.

The EOs (0.4 µg/µL) and their major components (0.2 µg/µL) were tested for their phytotoxicity against *Lolium perenne* and *Lactuca sativa* (monocotiledoneous and dicotiledoneous plants). Neither the EOs or their major components inhibited the germination of these plants at the end of the experiment (data not shown). The AasG EO inhibited the root and leaf growth of *L. perenne* (30% growth inhibition respect to the control), and stimulated the growth of *L. sativa root* (53% growth stimulation respect to the control.

The compound 1,8-cineole have reported phytotoxic effects against different plant species, including the moncot *Echinocloa crusgalli* and the dicot *Cassia obtusifolia* weeds, with the greatest effect on the monocot [31]; inhibited seed germination and seedling growth of *L. sativa* [32]; and inhibited the primary root growth of radish, *Raphanus sativus* [33]. This compound also inhibited all of the stages of mitosis in onion cells [32]. Camphor was phytotoxic against rice seedlings [34], and inhibited the seed germination and seedling growth of *L. sativa* [32] and the germination of radish [33]. Among the minor components of *A. assoana* EO, *p*-cymene was slightly active on *L. sativa* [32], and inhibited the root elongation of *Lepidium sativum* [33], and borneol, terpinen-4-ol, and linalool strongly inhibited seed germination and seedling growth of *L. sativa* [32]. Camphor, terpinen-4-ol, borneol, and linalool were present in higher concentrations in AasG EO (Table 1), and therefore could explain the phytotoxic effect of this oil.

When tested against *A. niger* spores, both EOs inhibited fungal germination at the maximum dose tested (Table 4), with the AasG EO being the most active (83% inhibition). The compounds 1,8-cineole and camphor, and their 1:1 combination, were not active against *A. niger* germination (Table 4). Among the minor components of the EO, *p*-cymene, linalool, α-pinene, α-, γ-terpinene, and terpinen-4-ol have reported antifungal actions, including synergism between 1,8 cineole and linalool (only present in the most active oil AasG) [35]. Therefore, the minor components of these EOs could play an important role in their antifungal effects. 

*A. pedemontana* subsp. *assoana* essential oils were not nematicidal, while the hydrolate resulted in a high mortality of J2 *M. javanica* (Table 5), which indicates that active components were present in the water fraction. The nematicidal activity of the hydrolate was further tested on the egg hatchability inhibition without any positive result (Table S2 in Supplementary Material), confirming that egg masses are less sensitive to the effects of extracts than J2 [11].

**Figure 2:**
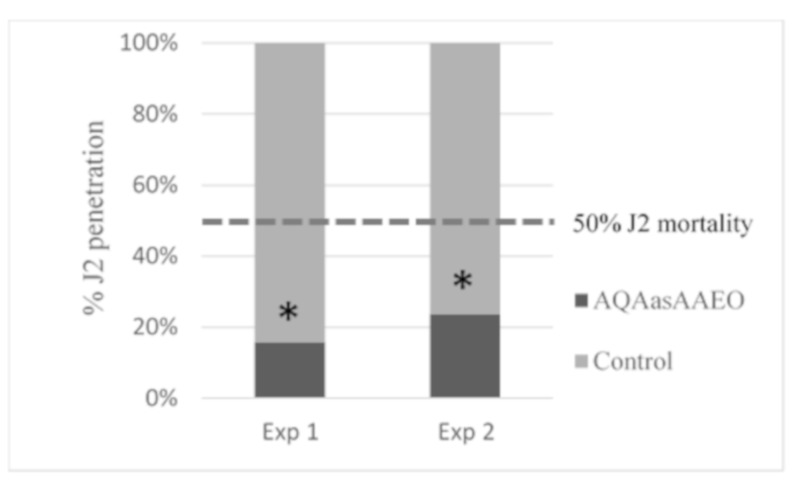
Effects of *Artemisia pedemontana* subsp. *assoana* hydrolate at a sublethal concentration (74%) on *Meloydogine javanica* juvenile infection capacity of *Solanum lycopersicum* root seedlings (two experiments). Bars represent the penetration percentage of treated J2 (hydrolate) vs. untreated J2 (control). * Denotes a statistically significant difference (Chi-square test) between the proportion of control juveniles (untreated) and treated juveniles that penetrated the tomato roots.

In vivo tests on tomato seedlings resulted in a strong suppression of the J2 root penetration when treated with the hydrolate at a sublethal dose (LD_50_; Table 5). The experiment was repeated twice, showing a significant decrease of J2 root penetration (34% and 26%) with respect to the control (inoculated with untreated J2; Figure 2). Therefore, the hydrolate affected the nematode behavior by suppressing juvenile penetration in the roots and reducing the nematode infection of tomato plants. 

Hydrolates are distillation byproducts, and contain volatile compounds with a high polarity that remain dissolved in water and do not occur in the essential oil [36]. Similar nematicidal effects have been reported for hydrolates from the distillation of *A. absinthium* var. Candial [11], *Lavandula x intermedia* var. super [37], *L. luisieri* [25,37], and two species of *Thymus* [37] against *M. javanica*. However, further research is needed in order to characterize the nematicidal components present in the active hydrolate of *A. pedemontana* subsp. *assoana*.

## 4. Conclusions

In summary, *A. pedemontana* subsp. *assoana* has been successfully cultivated in the greenhouse and in aeroponic cultivation. The EOs from greenhouse- and aeroponically-cultivated plants showed a 1,8-cineol and camphor chemotype with quantitative and qualitative chemical differences. These essential oils were trypanocidal, and showed moderate phytomonacidal effects. The EOs also had moderate insect antifeedant and antifungal effects, while the hydrolate was a strong nematicidal.

Both EOs were similarly antifeedant, while the EO from the greenhouse plants (flowering stage) was more biocidal (antifungal, nematicidal, and phytotoxic) than the EO from the aeroponic plants (growing stage), which was more antiparasitic. The major components of the oils (1,8-cineole and camphor) or their 1:1 combination did not explain any of these effects. We can conclude that these EOs have potential applications as insect antifeedants, and antifungal or antiparasitic agents, depending on the cultivation method, and if the hydrolate byproduct is a potent nematicidal.

## Figures and Tables

**Table 1:** Chemical composition of the essential oil (EO) from cultivated *Artemisia pedemontana* subsp. *assoana* (greenhouse—AasG; aeroponic—AasA).

Compounds	Rt ^a^	AasG	AasA
α-pinene	3.94	0.54 ^b^	0.89 ^b^
Camphene	4.16	1.77	5.34
β-pinene	4.56	0.22	0.71
α-Terpinene	5.15	-	0.99
*p*-Cymene	5.29	1.75	7.4
1,8-Cineole	5.43	22.88	25.78
γ-Terpinene	5.92	-	1.73
Sabinene	6.07	-	2.53
Linalool	6.66	1.56	-
Sabinene Isomer	6.68	-	1.45
Camphor	7.69	44.03	32.4
Borneol	8.12	4.79	2.77
Terpinen-4-ol	8.36	8.86	5.77
1-α-Terpineol	8.64	3.6	1.6
Bornyl acetate	10.77	0.98	-
Methyl eugenol	13.34	1.27	1.82
Nerolidol	16.78	0.82	-
Spathulenol	17.19	1.03	0.59
Viridiflorol	17.51	1.69	1.59

^a^ Retention time; ^b^ abundance: % area.

**Table 2:** Activity on *Trypanosoma cruzi* epimastigotes and *Phytomonas davidi* promastigotes of *Artemisia pedemontana* subsp. *assoana* EOs, their major components 1,8-cineole and camphor, and a 1:1 mixture of each compound.

EO/Compound	Concentration (µg/mL)	*T. cruzi* ^a^	*P. davidi* ^a^
AasG EO	800	100a	100
	400	100a	0.0
	200	20.3 ± 4.6b	0.0
AasA EO	800	99.4 ± 0.6a	100a
	400	100a	72.7 ± 12.9b
	200	72.1 ± 3.2b	0.0
	100	14.6 ± 2.9c	-
1,8-Cineole	100	2.0 ± 2.5	0.0
	10	0.0	0.0
Camphor	100	0.0	0.0
Cineole:Camphor 1:1	100	0.0	0.0

^a^ Data are expressed as % of growth inhibition (mean of three replicates ± standard error, relative to untreated controls). Values within the same column followed by a different letter are significantly different; one-way analysis of variance (ANOVA) with least significant difference test (*p* < 0.05) were used for the analysis.

**Table 3:** Insect antifeedant effects of *Artemisia pedemontana* subsp. *assoana* essential oils from greenhouse (AasG) and aeroponic plants (AasA); their major components, 1,8-cineole and camphor; and a 1:1 mixture of both compounds.

EO/Compound	Concentration (µg/cm^2^)	*S. littoralis*	*M. persicae*	*R. padi*
		%FI ^a^	%SI ^b^	%SI ^b^
AasG EO	100	85.6 ± 7.9 *	46.1 ± 8.9	56.9 ± 8.1 *
	50	43.3 ± 19.2	-	-
AasA EO	100	53.0 ± 8.9	76.1 ± 7.2 *	60.3 ± 7.2 *
	50	-	29.7 ± 7.6	-
1,8-Cineole	50	12.3 ± 7.6	42.1 ± 8.8	35.8 ± 7.4
Camphor	50	59.9 ± 12.6	32.3 ± 7.5	33.2 ± 7.8
Cineole:Camphor 1:1	50	62.7 ± 11.2	55.6 ± 8.4	40.4 ± 7.8

^a^ Percent feeding (%FI) inhibition. Values are means of 10 replicates ± standard error, ^b^ Percent settling (%SI) inhibition. Values are means of 20 replicates ± standard error, * *p* < 0.05, Wilcoxon signed-rank test.

**Table 4:** Effects of *Artemisia pedemontana* subsp. *assoana* essential oils; their major compounds, 1,8-cineole and camphor; and a 1:1 mixture of both compounds on *Aspergillus niger* spore germination.

EO/Compound	Concentration (µg/mL)	% I ^a^
AasG EO	800	83.7 ± 2.3a
	400	39.06 ± 6.3b
	200	33.9 ± 11.6b
AasA EO	800	67.8 ± 6.3a
	400	38.6 ± 13.4b
	200	16.1 ± 4.3c
1,8-Cineole	100	0.0
Camphor	100	24.2 ± 9.8
Cineole:Camphor 1:1	100	26.7 ± 8.0

^a^ Percent spore germination inhibition (I). Values within the same column followed by different letter are significantly different; one-way ANOVA with least significant difference (LSD) test (*p* < 0.05) were used for the analysis.

**Table 5:** Effects of *Artemisia pedemontana* subsp. *assoana* essential oils (EO) at 1 mg/mL and hydrolate (H) on mortality of *M. javanica* second stage juveniles (J2).

EO/H	J2 mortality (%) ^a^	LC50 ^b^
AasG EO	3.72 ± 0.23a	-
AasA EO	3.06 ± 0.35a	-
AasA H	96.4 ± 1.1b	74 (70–80)

^a^ Values are means of four replicates ± standard deviation, corrected according to Scheider–Orelli’s formula). Values within the same column followed by different letter are significantly different; one-way ANOVA with least significant difference (LSD) test (*p* < 0.05) were used for the analysis. ^b^ Five serial dilutions were used to obtain LC_50_ (95% confidence limits).

## Supplementary Materials

The following are available online at https://www.mdpi.com/2218-273X/9/10/558/s1, Table S1: Activity on FBIT test of *A. assoana* EOs, Table S2: Egg hatching inhibition effects of AasA hydrolate on *Meloidogyne javanica*.
