# Supplementary material for: Chemical Composition and Biological Activities of Artemisia pedemontana subsp. assoana Essential Oils and Hydrolate
Source: Biomolecules. 2019 Oct 2;9(10):558. doi: 10.3390/biom9100558 (PMC6843530; doi:10.3390/biom9100558)
Supplement: Supplementary file 1 [file biomolecules-09-00558-s001.pdf]

Supplementary Materials

Table S1 Activity on FBIT test of *A. assoana* EOs, the major compounds 1,8-cineole and camphor, and a mixture containing 50% of each compound.

Samples	Concentration (μg/mL)	FBIT (%Inhibition)
**AasW**	10000	0.0
	5000	0.0
	2500	0.0
**AasA**	10000	35.61±7.9
	5000	0.0
	2500	12.24±4.9
**1,8-Cineole**	100	0.0
	50	0.0
	25	0.0
**Camphor**	100	0.0
	50	0.0
	25	0.0
**1:1**	100	5.24±2.2
	50	6.81±2.85
	25	8.03±3.16
**Chloroquine diphosphate**	100	81.23±0.8
	50	85.32±0.7
	25	74.97±9.06
	12.5	45.39±14.9
	6.2	14.11±10.1
	3.1	9.64±3.1

Table S2 Egg hatching inhibition effects of AasA hydrolate on *Meloidogyne javanica* egg masses with time.

Treatment	Number of egg hatched[a] with time[b]				
	Day 0	Day 7	Day 14	Day 21	Day 28
**HaasA[c]**	128.5	318.5	228.7	21	0.25
**Control**	182.5	280.5	157.2	28.5	3

[a] Values are means of four replicates.[b]time 0: after 5 days of inmersion in test solutions; time 7 and subsequent times: number of days of inmersion in water after time 0.
[c]Undiluted hydrolate.
